# Temporal Trends in HAM/TSP incidence in Martinique over 25 years (1986-2010): dramatic decrease since 2000

**DOI:** 10.1186/1742-4690-12-S1-O6

**Published:** 2015-08-28

**Authors:** Stephane Olindo, Severine Jeannin, Martine Saint-Vil, Aissatou Signate, Mireille Edimonana-Kaptue, Julien Joux, Philippe Cabre, Raymond Cesaire, Agnes Lezin, Didier Smadja

**Affiliations:** 1 Service de Neurologie, Centre Hospitalier Universitaire de Martinique, CS 90632 -97 261 Fort-de-France Cedex, Martinique; 2 Service de Viro-Immunologie, Centre Hospitalier Universitaire de Martinique, CS 90632 -97 261 Fort-de-France Cedex, Martinique

## Background

Human T-Lymphotropic virus 1 (HTLV-1) has been discovered in 1980 and linked to tropical spastic paraparesis (HAM/TSP) in 1985 in Martinique. There is no data on HAM/TSP incidence trends. We report, in the present work, the temporal trends incidence of HAM/TSP in Martinique based on patients diagnosed in our unique Neurology Department in HAM/TSP diagnosis and management over 25 years. Methods: A registry has been set up since 1986 and HAM/TSP characteristics were collected carefully. All patients, living in Martinique, with a definite HAM/TSP onset between 1986 and 2010 were included in the present hospital-based study. The 25-year study time was stratified in five-year and ten-year periods. Crude incidence rates with 95% confidence interval (95%CI) were calculated using Poisson distribution for each period. The denominator was based on data provided by French National Institute for Statistical and Economic Studies (http://www.insee.fr) Results: Overall, 153 patients were identified (mean age at onset, 53+/-13.1 years; female: male ratio, 4:1). HAM/TSP incidence rates per 100,000 per 5 years (95%CI) in 1986-1990, 1991-1995, 1996-2000, 2001-2005 and 2006-2010 periods were 10 (6.8-7.3), 12.6 (9-16.1), 11.5 (8.1-14.9), 4.4 (2.5-6.4) and 2 (1.4-3.5). Between the two decades (1991-2000 and 2001-2010), ten-year HAM/TSP incidence rate decreased by 75% [19.1 (14.8-23.6) versus 4.9 (2.7-7.1)]. Age at onset did not differ between the different periods. Conclusion: There has been a significant decrease (75%) in HAM/TSP incidence in Martinique in the last decade. Prevention policies might have been effective in reducing HTLV-1 seroprevalence in our population. However, environmental factors such as parasite burden decrease could also account for HAM/TSP incidence dramatic fall.

